# Circulars and Bulletins from the Chief Surgeon’s Office

**Published:** 1918-09

**Authors:** 


### CIRCULARS AND BULLETINS FROM THE CHIEF SURGEON'S OFFICE

*War Medicine* publishes nothing of higher merit, from the scientific and literary viewpoint, than the Circulars and Bulletins prepared in the Chief Surgeon's office; and it could not carry to medical officers anything of more surpassing interest, or of greater importance to them, than the discussion of administrative and scientific problems in the Medical Department of the American Expeditionary Forces. Indeed, American medical officers cannot fail to feel a pardonable pride upon reading these official publications from the Chief Surgeon's Office, which show the really excellent work that is being carried on, and the high standards that are being maintained by those charged with protecting the health and the lives of the American soldiers.

These Circulars and Bulletins are not always published in their chronological order, nor are their numbers and dates given. Those of a confidential nature, and that contain information of interest only to relatively small groups of men are omitted. Sometimes one subject will be discussed in several numbers of the C. S. O. Circulars and Bulletins, in which case the paragraphs relating to one subject are grouped and published together. For instance, all of the references to promotion of medical officers in three Circulars are published together in this number.

Because of lack of space, all of the Circulars and Bulletins of a general nature, even though we realize that they would be of interest to medical officers, can not be published in *War Medicine*. Only the most important, and those of especial interest to all medical officers are selected. They are chosen not only because they represent the most authoritative discussion possible from the pens of many of the leading medical men in America, both from military and civil life, but because, from the journalistic point of view, they present high-class medical literature on timely subjects that would appeal most to the interest of the physicians who are engaged with the problems of the medicine, surgery and hygiene of the war.

These extracts from the Circulars and Bulletins from the Chief-Surgeon's Office are indexed according to subjects, and the medical officers who keep their copies of *War Medicine* will thus have a permanent record of many of the most important documents that are prepared for their use. Reference to the files of Division Surgeons, and of Commanding Officers of Hospitals and other Units, will locate the number and dates of the mimeographed Circulars and Bulletins relating to the subjects dealt with in the extracts or excerpts from them that are published in *War Medicine*.

Editorial comment on the Circulars relating to the new plan for the promotion of medical officers is made in this number of *War Medicine*. Among other administrative matters dealt with in the C. S. O. Circulars, which are reproduced, and which are of interest to all medical officers, may be mentioned the plan of organization of Division Laboratories and Infectious Diseases, and the activities of the various branches of medicine embraced in that Division; the prophylactic use of anti-tetanic serum; and the administration of messes.

The excerpts from the Bulletins on Diseases from the Chief

Surgeon's Office in this number are timely and of much importance to medical officers generally. A note of passing interest is an observation on "three days' fever" or "Spanish Flu", which has been proved by the bacteriologist of the American Expeditionary Forces to be due to the Pfeiffer bacillus, and therefore the methods of prevention are the same as those employed in other epidemics of influenza.

With the military activities necessary to drive the Huns back into Germany, we shall expect many casualties among our soldiers, and therefore the brief articles extracted from Bulletins on shock and gas prevention extracted from the Bulletins of Diseases present absorbing topics that are dealt with in a very practical manner.

The article extracted from two numbers of the Bulletin of Diseases, entitled "A System of Venereal Prophylaxis", p. 283 in which the dignity of the "Professor of Prophylaxis" is upheld, and which contains a full description of the operative technic that has been worked out for the administration of venereal prophylaxis, is really medical literature of serious importance. If this "system" is followed throughout the army, it will aid in reducing the non-effective rate among our soldiers, and thereby increase the force and fighting efficiency of our troops.

